# Prediction of calcium-binding sites by combining loop-modeling with machine learning

**DOI:** 10.1186/1472-6807-9-72

**Published:** 2009-12-11

**Authors:** Tianyun Liu, Russ B Altman

**Affiliations:** 1 Department of Genetics, Stanford University, Stanford, CA, USA; 2 Department of Bioengineering, Stanford University, Stanford, CA, USA

## Abstract

**Background:**

Protein ligand-binding sites in the apo state exhibit structural flexibility. This flexibility often frustrates methods for structure-based recognition of these sites because it leads to the absence of electron density for these critical regions, particularly when they are in surface loops. Methods for recognizing functional sites in these missing loops would be useful for recovering additional functional information.

**Results:**

We report a hybrid approach for recognizing calcium-binding sites in disordered regions. Our approach combines loop modeling with a machine learning method (FEATURE) for structure-based site recognition. For validation, we compared the performance of our method on known calcium-binding sites for which there are both holo and apo structures. When loops in the apo structures are rebuilt using modeling methods, FEATURE identifies 14 out of 20 crystallographically proven calcium-binding sites. It only recognizes 7 out of 20 calcium-binding sites in the initial apo crystal structures.

We applied our method to unstructured loops in proteins from SCOP families known to bind calcium in order to discover potential cryptic calcium binding sites. We built 2745 missing loops and evaluated them for potential calcium binding. We made 102 predictions of calcium-binding sites. Ten predictions are consistent with independent experimental verifications. We found indirect experimental evidence for 14 other predictions. The remaining 78 predictions are novel predictions, some with intriguing potential biological significance. In particular, we see an enrichment of beta-sheet folds with predicted calcium binding sites in the connecting loops on the surface that may be important for calcium-mediated function switches.

**Conclusion:**

Protein crystal structures are a potentially rich source of functional information. When loops are missing in these structures, we may be losing important information about binding sites and active sites. We have shown that limited loop modeling (e.g. loops less than 17 residues) combined with pattern matching algorithms can recover functions and propose putative conformations associated with these functions.

## Background

Calcium is one of the most important metals for life. Calcium-binding sites in proteins play a wide range of roles including stabilizing protein structures or acting as cofactors in catalytic and regulatory processes[1]. The most common configuration of calcium-binding sites is the EF-hand motif. Other types of calcium-binding sites show great variation in coordinating residues, sizes and structural motifs. Predicting and identifying calcium-binding sites in proteins are essential for understanding the roles of calcium in biological systems. Unfortunately, there are no universally applicable sequence motifs for calcium binding, and so they are best recognized with structure-based methods[2].

Existing methods for binding sites recognition can be classified into four types: (1) homology based annotation transfer methods (2) geometric methods, (3) energetic methods, and (4) methods using other criteria, including the characteristic distribution of chemical properties, the 3D motif, the surface accessibility, and the stability of proteins[3,4]. We have previously presented a knowledge-based method, FEATURE, which calculates physical and chemical properties in concentric shells around a potential binding site, with a Bayesian score to predict its likelihood of ligand binding[5]. FEATURE has been applied to predict sites such as calcium-binding, zinc-binding, ATP-binding and others[6,7]. One advantage of FEATURE is its sequence independence and thus it is able to recognize divergent binding sites based on the three-dimensional configuration of atoms in these sites.

FEATURE has been applied to both static crystal structures as well as to multiple conformations generated by short (1 nanosecond) molecular dynamics simulations[8]. FEATURE has better performance in identifying calcium-binding sites when it is applied to multiple configurations produced by dynamic simulations. These results underscore the dynamic properties of many ligand-binding sites, including calcium. Indeed, residues that coordinate a metal often undergo conformational changes upon binding[9]. Calcium binding often results in conformational changes and an increase in the stability of proteins (as is the case, for calmodulin and troponin C[10]. Unfortunately, in the absence of ligands, binding sites in their apo states are often disordered and their 3D structures cannot be determined experimentally. In this work, we refer to regions for which 3D coordinates are not present in the Protein Data Bank (PDB) file as "unstructured" regions. More than 2/3 of the crystal structures in the PDB contain unstructured regions[11]. This frustrates the recognition of ligand-binding sites by methods, which depend on a 3D structure.

In this study, we combine previously published loop modeling methods with FEATURE to predict calcium-binding sites in unstructured regions for which there is no crystallographic electron density [12-14]. The loop building and calcium recognition codes have both been validated separately. The contribution of this paper is to combine them and show useful results in recovering calcium binding sites in loops as long as 17 residues. We first validate the method by testing its performance on a set of calcium binding sites for which there is both a holo structure (calcium bound) and an apo structure (calcium missing) available in the PDB. Many of the calcium-binding loops in the crystallographic apo structures do not adopt recognizable calcium-binding configurations. However, when we rebuild them, the calcium binding sites become clear. We then go on to predict calcium binding in a set of rebuilt loops. We predict calcium-binding sites that achieve high FEATURE scores and further characterize these novel predictions, a subset of which we can validate using the literature.

## Results

### FEATURE scan of validation dataset

We are specifically targeting calcium ions that are coordinated by atoms in the protein structure and are not on the surface mediated by water. We anticipate that sites mediated by protein atoms are more likely to be specific. It would be interesting and significantly more challenging to predict calcium sites whose binding is mediated in large part by water molecules. Using the 56 sites published in Babor's work we derived a dataset of positive sites contains 20 calcium-binding loops of which structures of loop regions in both the apo and the holo forms have been resolved using X-ray diffraction (Table 1)[9].

**Table 1:** 20 apo-holo pairs of calcium-binding proteins for validation

		Holo structure		Apo structure			Loop			
				
Protein and calcium ID	Protein name	PDB ID	Chain ID	PDB ID	Chain ID	All RMSD (Å)	Start	End	RMSD (Å)	Sequence
1B9AA110	PARVALBUMIN	1B9A	A	1B8C	A	1.48	51	62	2.68	AQDKSGFIEEDE

1C1R_247	CHYMOTRYPSIN INHIBITOR 2	1C1R	A	1AZ8	A	0.60	70	80	0.13	EDNINVV**E**GN**E**

1DPO_246	TRYPSIN	1DPO	A	1BRB	E	0.73	70	80	0.37	**E**HNINVL**E**GN**E**

1DVIB275	CALPAIN	1DVI	B	1AJ5	B	2.06	111	121	2.36	AG**DD**M**E**VSAT**E**

1DVIB277	CALPAIN	1DVI	B	1AJ5	A	1.75	184	195	0.90	**D**T**D**RSGTIGSN**E**

1F6SB202	ALPHA-LACTALBUMIN	1F6S	B	1F6R	C	0.86	79	89	0.33	KFL**DDD**LT**DD**I

1HAZB1246	BETA-CASOMORPHIN-7	1HAZ	B	1FLE	E	1.06	70	80	0.23	**E**HNLNQNNGT**E**

1I40_305	INORGANIC PYROPHOSPHATASE	1I40	A	1MJW	A	0.55	138	146	0.25	F**E**HYK**D**L**E**K

1K94_998	GRANCALCIN	1K94	A	1F4Q	B	1.23	130	143	0.68	TVDQDGSGTV**E**HH**E**

1K94_999	GRANCALCIN	1K94	A	1F4Q	A	1.35	62	72	0.67	AGQDG**E**V**D**A**EE**

1K96A91	S100A6	1K96	A	1K8U	A	4.15	24	34	1.45	G**D**KHTLSKK**E**L

1KXQB4003	ALPHA-AMYLASE, PANCREATIC	1KXQ	B	1KXV	A	0.46	165	172	0.17	LL**D**LAL**E**K

1NLS_240	CONCANAVALIN A	1NLS	A	1DQ0	A	0.31	9	20	0.47	L**D**TYPNT**D**IG**D**P

1NOL_632	HEMOCYANIN (SUBUNIT TYPE II)	1NOL	A	1OXY	A	0.55	577	584	0.13	V**D**AVSYCG

1PSH_1	PHOSPHOLIPASE A2	1PSH	A	1A3D	A	0.61	25	34	1.41	GCYCGRGGSG

1QMDA403	PHOSPHOLIPASE C	1QMD	A	1QM6	A	0.20	265	270	0.13	SG**E**K**D**A

1QMDA405	PHOSPHOLIPASE C	1QMD	A	1QM6	A	0.20	292	299	0.14	M**D**NPGN**D**F

2POR_304	PORIN	2POR	A	3POR	A	1.00	135	145	0.98	S**D**GKVG**E**TS**ED**

3LHM_131	HUMAN LYSOZYME	3LHM	A	2LHM	A	0.21	83	94	0.40	ALL**DD**NIA**DD**VA

5CHY_401	CHEY	5CHY	A	3CHY	A	0.35	55	63	0.15	IS**D**WNMPNM

PDB ID (PDB code followed by chain identifier and calcium index) and protein names are from PDB (column 1-2). PDB ID and chain ID are from PDB (column 3-6). The RMSD between the apo and the holo structures is given in column 7. Columns 8, 9 and 11 list the information of calcium-binding loops, including the loop starting and ending residue numbers, and the loop sequence. The RMSD between calcium-binding loops in the apo and the holo structures is given in column 10.

Table 2 shows the performance of FEATURE on the holo structures, the apo structures, the apo structures of which the binding loops were removed (apo-gap), and the apo structures for which the binding loops (apo-loop) were rebuilt using modeling methods (See Method section: Construction of loop structures). The accuracy of FEATURE recognizing calcium-binding site has been benchmarked via cross-validation and independent test sets and the specificity on the test set is above 99% at a score cutoff of 50. Using this cutoff, FEATURE recognizes 15 out of 20 calcium-binding sites in the holo structures [5,15,16]. (High specificity guarantees a low rate of false positives, at the expense of sensitivity, which reflects false negatives. In the set, we have five false negatives.) The predicted sites are about 0.0 Å to 2.5 Å away from the experimentally observed site. Combined with modeling methods, FEATURE recognizes 14 out of 20 calcium-binding sites in the apo-loops. For comparison, FEATURE identifies binding sites correctly in only 7 out of the 20 apo structures, and 0 out of the 20 apo-gaps. Table 2 also compares the highest FEATURE scores of the apo structure and the apo-loop. Of the 20 pairs, 17 apo-loops have higher FEATURE scores than the corresponding apo structures.

**Table 2:** FEATURE benchmark of the 20 apo-holo pairs of calcium-binding proteins

Protein and calcium ID	Holo structure	Apo structure	Apo-gap	Apo-loop
1B9AA110	**81.72**	26.75	5.41	**51.58**

1C1R_247	**56.57**	**51.71**	1.94	**55.43**

1DPO_246	**52.89**	47.94	-0.17	**50.87**

1DVIB275	**66.89**	10.63	0.00	29.70

1DVIB277	**68.39**	**57.51**	-18.90	41.18

1F6SB202	**68.11**	**54.04**	16.58	**54.46**

1HAZB1246	**51.82**	48.21	-4.82	32.72

1I40A305	30.52	27.87	16.92	46.64

1K94_998	**80.71**	**63.46**	-22.81	42.92

1K94_999	40.31	32.63	-0.45	**50.96**

1K96A91	**79.61**	45.63	9.77	**59.27**

1KXQB4003	**54.99**	43.25	22.01	**59.53**

1NLS_240	**61.98**	**52.09**	28.46	**60.76**

1NOL_632	36.88	30.22	30.22	41.85

1PSH_1	34.09	33.81	22.03	**56.37**

1QMDA403	**60.45**	30.41	27.37	**50.41**

1QMDA405	**59.81**	45.00	37.05	**52.09**

2POR_304	47.41	**71.34**	34.96	**89.29**

3LHM_131	**61.69**	**54.34**	2.46	**58.95**

5CHY_401	**50.19**	46.70	23.56	**54.08**

We compare the performance of FEATURE on the holo structures, the apo structures, the apo structures of which the binding loops were removed (apo-gap), and the apo structures of which the binding loops (apo-loop) have been rebuilt using modeling methods. Protein and calcium ID are from PDB (column 1). FEATURE scores that evaluate the probability of the likelihood of a site being calcium-binging are listed in column 2-5. Of the 20 pairs, 17 apo-loops have higher FEATURE scores than the corresponding apo structures. Bolded number indicates that FEATURE identifies the calcium-binding site correctly using a score threshold of 50. FEATURE identifies 15 out of 20 calcium-binding sites in the holo structures. Combined with modeling methods, FEATURE identifies 14 out of 20 calcium-binding sites in the apo-loops. For comparison, FEATURE identifies binding sites correctly in only 7 out of 20 the apo structures, and 0 out of 20 the apo-gaps. These results demonstrate that reconstruction of the calcium-binding loops in the apo structures allows FEATURE to identify cryptic calcium sites effectively.

These results demonstrate that reconstruction (and generation of structural diversity) of the calcium-binding loops in the apo structures allows FEATURE to identify cryptic calcium sites effectively.

As a negative control, we created a dataset of nonsites using the 20 pairs of apo and holo structures. The negative control contains a set of random selected loops (excluding the calcium-binding loop) in the holo structures and the counterpart in the apo structures. The local environments surrounding nonsites never achieves scores compatible with calcium binding. The score of rebuilt loops (local environments) ranges from -39.9 to 45.6, indicating that the score cutoff of 50 is, indeed, highly specific (low false positive rate) [15-17].

**Figure 1:**
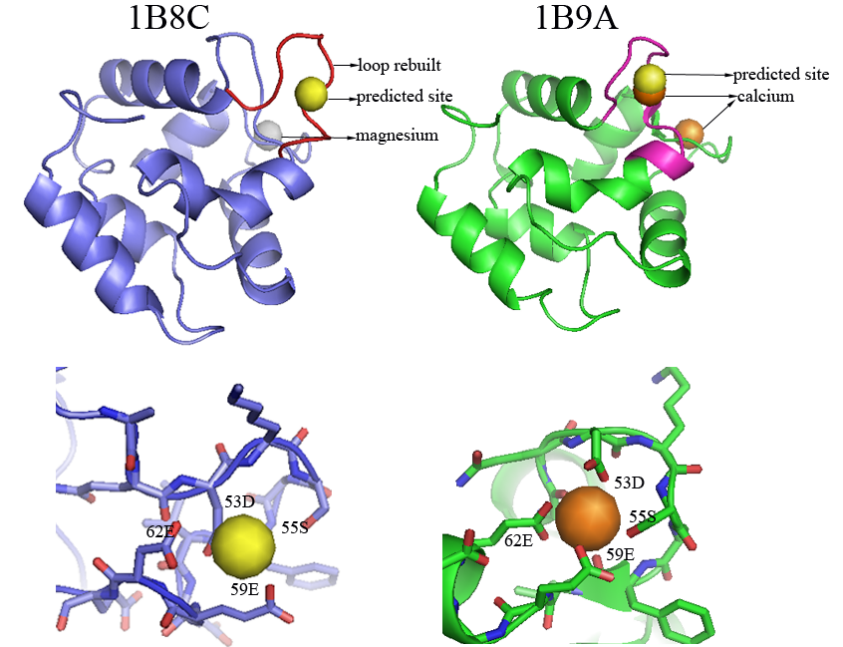
**An example illustrating a successful FEATURE prediction with the help of modeling methods**. Parvalbumin-beta from *cyprinus carpio *is a calcium-binding protein and two structures 1B8C and 1B9A have been resolved experimentally. 1B9A binds to two calcium ions via loop 90-97 (residue 90-97) and loop 51-62 (pink, residues 51-62: AQDKSGFIEEDE). 1B8C binds to one magnesium ion via loop 90-97. The loop 51-62 in 1B8C does not bind any metal ions, thereby considered as apo form. The RMSD between 1B9A and 1B8C is 1.48 Å. The RMSD of loop 51-62 in 1B9A and 1B8C is 2.68 Å. By scanning loop 51-62, FEATURE successfully identifies the sites in 1B9A, but not in 1B8C. In 1B8C, FEATURE can identify the site only when the loop 51-62 is rebuilt using modeling methods. The close-up view shows (red for oxygen atoms) that the predicted site in 1B8C and the experimentally observed site in 1B9A are similar. Both sites are in close associations with oxygen atoms from four residues 53D, 55S, 59E and 62E. This example demonstrates that FEATURE can successfully identify calcium-binding sites in holo structures and in apo structures with binding loops rebuilt by modeling methods.

Figure 1 shows an example of how modeling methods improve the performance of FEATURE. Parvalbumin-beta (from *cyprinus carpio*) is a member of EF-hand family. Two structures have been resolved experimentally, 1B9A and 1B8C[18]. The all-atom root mean square deviation (RMSD) between 1B9A and 1B8C is 1.48 Å. 1B9A binds to two calcium ions via loop 90-97 (residue 90-97) and loop 51-62 (residues 51-62: AQDKSGFIEEDE). 1B8C binds to one magnesium ion via loop 90-97. The loop 51-62 in 1B8C does not bind any metal ions, thereby considered as apo form. The RMSD of loop 51-62 in 1B9A and 1B8C is 2.68 Å. By scanning loop 51-62, FEATURE successfully identifies the sites in 1B9A, but not in 1B8C. Using modeling methods, an alternative structure of loop 51-62 in 1B8C was rebuilt. In the presence of this rebuilt calcium-binding loop, FEATURE identifies the calcium-binding site correctly. The predicted site in 1B8C and the experimentally observed site in 1B9A are similar. Both sites are in close associations with oxygen atoms from four residues 53D, 55S, 59E and 62E.

In summary, applying FEATURE and modeling methods to the 20 apo-holo pairs of calcium-binding proteins demonstrates that our procedure can identify calcium-binding sites successfully even when structures of binding-loops are not well structured, and gives us confidence to make novel predictions. Importantly, these predictions have a low positives (but importantly, not zero) likelihood of being false, based on both our previous validations and our tests on these 20 pairs. We thus are confident that we can seek novel calcium binding sites.

### Predict calcium-binding sites in proteins of which structures of the binding loops have not been resolved experimentally

We scanned all proteins that share SCOP folds families with known calcium-binding proteins. The candidate dataset consisting of 2030 PDB chains containing 2745 structural gaps. We built structures for the 2745 gaps using modeling methods. FEATURE predicts calcium binding in the loop structures for 256 of these gaps, from which 102 non-redundant predictions were derived. These 102 predictions were further divided into three groups (Table 3, 4 and 5, Additional file 1: Table S1) according to the availability of experimental evidence: direct evidence in the literature, indirect evidence from similar known calcium-binding proteins, and truly novel predictions.

**Table 3:** Ten FEATURE predictions confirmed by direct experimental evidence.

		Loop				FEATURE score	
				
Protein name	Protein ID Chain ID	Start	End	Sequence	SiteDis (Å)	Gap	Loop
VITAMIN B12 RECEPTOR	1NQG_A	231	241	AYYSPGSPLL**D**	2.06	42.41	91.49

BEAN LECTIN-LIKE INHIBITOR	1DHK_B	89	96	VQP**E**SKG**D**	3.40	27.35	61.10

Two predictions of which calcium binding have been observed experimentally near the predicted sites. Column 2 lists protein name that is PDB code followed by the chain identifier. We build structures for gaps of which 3D structures are not presented in the original PDB files. Information of these structures, referred as "loop", is given in column 3-5. Column 6 lists the distances between the predicted sites and the experimentally observed sites (SiteDis). Column 7 and 8 list FEATURE scores at the predicted sites in the structures where loop structures are missing (Gap) and the structures of which loop structures were rebuilt by modeling methods (Loop). These two are holo structures, but their calcium-binding sites are not completely structured. FEATURE is able to identify these sites in the presence of the rebuilt loop structures.

**Table 4:** Eight predictions of which their holo structures have been identified through a homologous searching process using MAMMOTH

Protein name	PDB ID	Loop				FEATURE score		Holo structure				Alignement		
				
		Start	End	Sequence	CaDis(Å)	Gap	Loop	SCOP ID	Start	End	CaDis(Å)	PRA	RMSD	PID
CONCANAVALIN A	1APN_A	15	23	T**D**IG**D**PSYP	2.40	9.15	58.19	d1vald_	15	23	1.94	99	1.61	96

C-REACTIVE PROTEIN	1LJ7_H	142	149	FGGNF**E**GS	1.94	-18.56	55.46	d1gnhc_	142	149	1.99	100	0.74	100

SOLUBLE INORGANIC PYROPHOSPHATASE	1IPW_A	97	101	**DE**AG**E**	4.67	47.13	53.38	d1i6ta_	97	101	2.19	99	1.49	91

B**E**TA-KETOACYL REDUCTASE	1I01_C	140	149	VGTMGNGGQA	2.25	7.93	56.74	d1q7bd_	140	149	2.41	96	1.96	89

OBELIN	1SL9_A	122	128	F**D**K**D**GSG	1.96	-1.98	50.80	d1sl7a_	122	128	2.43	98	2.89	89

DEOXYRIBONUCLEASE I	2DNJ_A	99	107	**D**GC**E**SCGN**D**	2.07	42.69	72.61	d3dnia_	99	107	2.57	100	0.87	98

STAPHYLOCOCCAL NUCLEASE	1SNQ_A	44	51	TKHGKKGV	4.17	45.69	66.14	d1nuca_	44	51	4.88	98	1.70	92

SOLUBLE INORGANIC PYROPHOSPHATASE	1FAJ_A	145	149	**E**KGKW	3.35	39.95	51.60	d1i40a_	146	149	5.37	99	1.59	87

Column 2 lists protein name that is PDB code followed by the chain identifier. We build structures for gaps of which 3D structures are not present in the original PDB files. Information of these structures, referred as "loop", is given in column 2-5. Column 5 lists the distances from the predicted sites to the rebuilt loops (CaDis). Column 6 and 7 list FEATURE scores at the predicted sites in the structures where loop structures are missing (Gap) and in the rebuilt loop structures (Loop). Ligands observed experimentally are listed in column 8 and the ligand IDs are from PDB. Column 9 shows the SCOP ID of the holo structures. Information of aligned counterparts for the binding loops is listed in column 10-12. Column 12 lists the distances from the calcium ions to the aligned loop counterparts (CaDis). The percentage of residues aligned (PRA), the RMSD of the aligned regions, and the percentage of identical residues (PID) are shown in column 13-15.

**Table 5:** 14 FEATURE predictions of which experimentally solved homologous holo structures are found through a homologous searching process using MAMMOTH

	Loop				FEATURE score			Holo structure				Alignement			
					
PDB ID	Start	End	Sequence	CaDis(Å)	Gap	Loop	Ligand	SCOP ID	Start	End	Sequence	CaDis(Å)	PRA	RMSD	PID
1G57_B	33	39	**DDED**R**E**N	2.29	-29.15	62.92	CS	d1pvwa_	21	27	**D**S**DE**R**E**G	2.11	86	2.65	24

1VJS_A	181	193	AW**D**W**E**VSN**E**NGNY	2.49	41.99	82.70	None	d1e40a2	178	193	**E**GKAW**D**W**E**VSS**E**NGNY**D**YLMY	2.18	97	1.06	66

2A9Q_A	53	59	LMLP**E**I**D**	3.34	44.10	57.51	None	d1zh2b1	53	59	LGLP**D**G**D**	2.23	98	1.34	41

1GIH_A	149	164	ARAFGVPVRTYTH**E**VV	3.16	37.28	60.16	1PU	d1u5ra_	173	184	ASIMAPANFVG	2.47	81	3.03	19

1OZT_G	127	141	GKGGN**EE**STKTGNAG	1.71	23.67	61.66	None	d1sxsb_	125	139	GRGGN**EE**STKTGNAG	3.53	87	1.86	71

1LEW_A	173	183	RHT**DDE**MTGYV	2.20	10.16	71.76	None	d2eufb1	170	180	YSFQMALTSVV	4.18	85	2.92	25

2JAV_A	131	141	S**D**GGHTVLHR**D**	2.60	8.93	56.86	5Z5	d2auha1	1123	1132	LNAKKFVHR**D**	4.87	85	3.06	16

2ANT_I	29	43	KAT**EDE**GS**E**QKIP**E**A	2.23	20.71	80.94	NAA	d1jmja_	68	99	S**EDD**LQLFH	5.09	92	2.31	26

1U5I_A	563	568	KR**ED**IK	2.97	39.39	66.52	None	d1alwb_	127	133	TRHP**D**LK	5.37	98	1.82	13

1EB7_A	222	229	ASVLPSG**D**	3.96	19.22	58.45	CA,HEC	d1iqca2	207	213	**E**TKNPAA	6.58	86	1.79	21

1ANT_L	395	406	LNPNRVTFKANR	2.95	42.78	77.77	None	d1jmja_	446	454	TQVRFTV**D**R	6.64	88	2.72	26

2IYN_C	84	97	ARG**EEED**RVRGL**E**T	3.26	35.10	52.26	MG	d1srrc_	81	96	MTAYG**E**L**D**MIQ**E**SK**E**L	6.88	96	2.7	25

2IK4_A	103	113	PNVSHP**E**TKAV	3.95	41.81	64.20	MG,PO4,	d1i40a_	59	63	NHTLS	4.03	91	3.49	13

Column 1 lists PDB code followed by the chain identifier. We build structures for gaps of which 3D structures are not present in the original PDB files. Information of these structures, referred as "loop", is given in column 2-5. Column 5 lists the distances from the predicted sites to the rebuilt loops (CaDis). Column 6 and 7 list FEATURE scores at the predicted sites in the structures where loop structures are missing (Gap) and in the rebuilt loop structures (Loop). Ligands observed experimentally are listed in column 8 and the ligand IDs are from PDB. Column 9 shows the SCOP ID of the homologous holo structures. Information of aligned counterparts for the binding loops is listed in column 10-13. Column 13 lists the distances from the calcium ions to the aligned loop counterparts (CaDis). The percentage of residues aligned (PRA), the RMSD of the aligned regions, and the percentage of identical residues (PID) are shown in column 14-16.

We have found direct experimental verifications for ten (~10%) of the 102 non-redundant predictions (Table 3 and 4). Table 3 shows the two predictions in which calcium binding have already been observed experimentally near our predicted sites. They are 1NQG chain A residues 231-241 and 1DHK chain B residues 89-96. In these two proteins, partial 3D structures for the predicted sites are available. After rebuilding the binding sites using modeling methods, FEATURE identifies these two sites. Table 4 shows eight predictions of which their holo structures have been identified through a homologous searching process by using program MAMMOTH[19]. The structural homologs of the eight predictions satisfy three conditions: (1) the percentage of residue aligned (PRA) of the structural alignments between the prediction and the corresponding homologous structures is higher than 95%, (2) the percentage of identical residues (PID) is higher than 85% (considering sequence shift in structural alignments) and (3) the distances between the calcium-binding loop in the homologous counterpart and calcium ions (CaDis) is shorter than 5.4 Å. These matched homologous structures are essentially holo structures for our targets. Interestingly, in some cases, the loop does not directly interact with the calcium ion, but residues in the loop contribute to the FEATURE score. For example, the last row in Table 4, the minimum distance between the  predicted site to the loop (residue 145-149) is 5.37 Å. The predicted site directly coordinates with D65, D67, D97 and D102. The residue K148 actually provides positive balance charges for the sites. The coordination of the predicted site in 1FAJ is the same as that in the holo structures 1I40.

**Figure 2:**
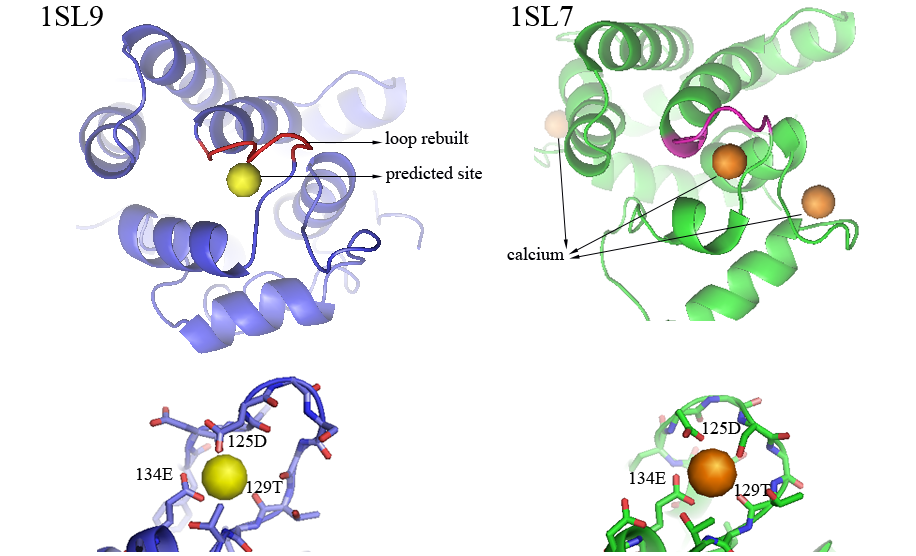
**FEATURE predictions confirmed by experimentally solved structures**. The structure of obelin from *obelia longissima *in its apo form (PDB ID: 1SL9) is shown in blue. In the original structure, loop 122-128 (residues 122-128, sequence: FDKDGSG) is unstructured. The structure of this region was built using modeling methods. FEATURE predicts a calcium-binding site in 1SL9 in the presence of the structure rebuilt for loop 122-128. The predicted site adopts an EF-hand motif. Searching for homologs, we found that the calcium-binding form obelin has been resolved experimentally (PDB ID: 1SL7). The predicted binding site in 1SL9 is similar with one of the three calcium-binding sites observed in 1SL7. Both sites are in close association with oxygen atoms (red in close-up view) from residues 125D, 129T and 134E. The RMSD between 1SL9 and 1SL7 is 3.01 Å and that between the rebuilt loop in 1SL9 and the counterpart in 1SL7 (pink) is 2.27 Å.

Figure 2 shows the structure of obelin from *obelia longissima *in its apo form (PDB ID: 1SL9), which has a structural gap at residues 122-128 (sequence: FDKDGSG) [20]. When the structure for the gap is rebuilt using modeling methods, FEATURE identifies a calcium-binding site near the gap. The predicted site adopts an EF-hand motif according to the SCOP fold topologies. Searching for homologs, we found that the calcium-binding form obelin has been resolved experimentally (PDB ID: 1SL7). The RMSD between 1SL9 and 1SL7 is 3.01 Å and that between the rebuilt loop in 1SL9 and the counterpart in 1SL7 is 2.27 Å. This new apo-holo pair was not contained in our validation set. The predicted binding site in 1SL9 is similar with one of the three calcium-binding sites observed experimentally in 1SL7.

**Figure 3:**
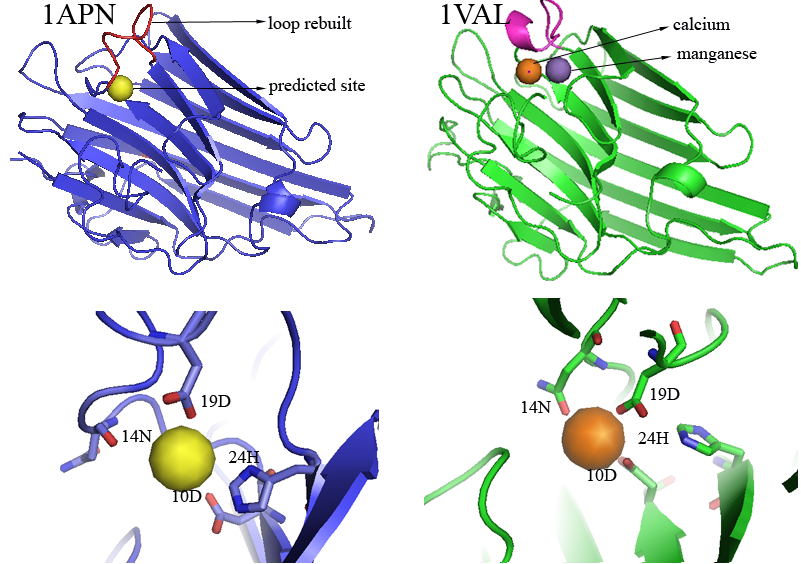
**FEATURE predictions confirmed by experimentally solved structures**. The structure of concanavalin A from *canavalia ensiformis *in its apo form (PDB ID: 1APN) is shown in blue. In the original apo structure, loop 15-23 (residues 15-23, sequence TDIGDPSYP) is unstructured. The structure of loop 15-23 was rebuilt using modeling methods. FEATURE predicts a calcium-binding site in 1APN in the presence of the structure of loop 15-23. Searching for homologs, we found that the calcium-binding form concanavalin A has been resolved experimentally (PDB ID: 1VAL). 1VAL binds to a calcium ion and a manganese ion. The close-up view (red for oxygen atoms) shows that the predicted site is similar with the calcium-binding site in 1VAL identified experimentally. The comparison also suggests that more than one metal ions binds to the predicted site. The RMSD between 1APN and 1VAL is 1.61 Å and that between the rebuilt loop in 1APN and the counterpart in 1VAL (pink) is 3.23 Å.

Another example (Figure 3) is the apo structure of concanavalin A from *canavalia ensiformis *(PDB ID: 1APN), of which loop 15-23 (residues 15-23, sequence TDIGDPSYP) is unstructured[21]. A blind scan for calcium-binding sites in 1APN failed because loop 15-23 is unstructured. However, FEATURE identifies this calcium-binding site correctly when the structure of loop 15-23 is rebuilt using modeling methods. Searching for homologs, we found that the calcium-binding form concanavalin A has been resolved experimentally (PDB ID: 1VAL) [22,23]. In the structure of 1VAL, four residues 10D, 14N, 19D and 24H coordinate a calcium ion and a manganese ion, but residue 19D cannot be located in the initial apo structure (1APN). The close-up view shows that the predicted site is similar with the calcium-binding site in 1VAL identified experimentally. The comparison also suggests that more than one metal ions bind to the predicted site.

The predictions in Table 3 and 4, Figure 2 and Figure 3 show the rediscovery of calcium-binding sites and they demonstrate our method's effectiveness.

We have also found indirect experimental verifications for 14 (14%) of the 102 non-redundant predictions. Table 5 shows the 14 predictions for which homologous holo structures have been identified (See Method section: Validation of predictions). The structural homologs of the 14 predictions satisfy two conditions: (1) the PRA of the structural alignments between the prediction and the corresponding homologous structures is higher than 80% and (2) the CaDis is shorter than 6.9 Å. Further visual inspection confirms that the manner of calcium binding in the structural homologs and that in the predicted sites is similar.

**Figure 4:**
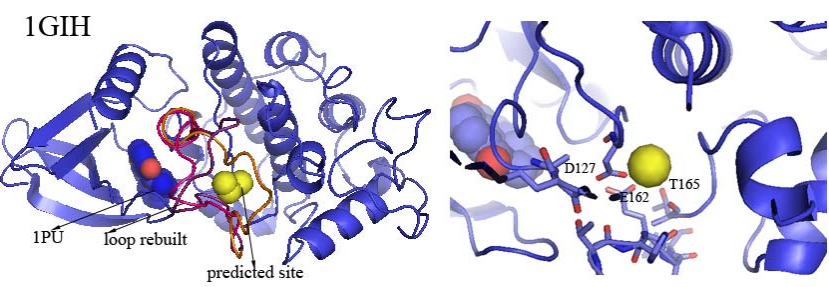
**The predicted calcium-binding site in the structure of human cell division protein kinase 2 **(PDB ID: 1GIH)** and the experimentally observed site in the structural homolog of **1GIH. 1GIH** binds to 1PU (sphere colored by element, gray blue for carbon, dark blue for nitrogen, and red for oxygen)**. The structure of one large loop 149-164 (residues 149-164, sequence: ARAFGVPVRTYTHEVV) near the 1PU binding site is unstructured. Three possible loop conformations built by modeling methods are shown in red, pink and orange. The rebuilt loops locate at the domain interface and cover the binding pocket of 1PU. FEATURE identifies a calcium-binding site in the presence of the rebuilt loops. The close-up view shows the close associations between the predicted site and three residues F127, E162 and T165. The predicted site is 13 Å away from 1PU.

Figure 4 shows an example of predictions for which homologous holo structures have been identified. The human cyclin-dependent protein kinase 2 (CDK2) (PDB ID: 1GIH) binds to 1PU (1-(5-OXO-2,3,5,9B-TETRAHYDRO-1H-PYRROLO [2,1-A]ISOINDOL-9-YL)-3-PYRIDIN-2-YL-UREA) [24]. The structure of one large loop 149-164 (residues 149-164, sequence: ARAFGVPVRTYTHEVV) near the 1PU binding site is disordered and its 3D coordinate are not present in the original PDB file. FEATURE predicts a calcium-binding site in the modeled loop. Figure 4 shows three possible loop conformations in red, pink and orange in the left panel. In particular, these rebuilt structures of the loop149-164 occur at the domain interface and cover the binding pocket of 1PU, suggesting that calcium may affect 1PU binding by changing the conformation of the loop and the domain interface. We have studied 30 structures of CDK2. These structures bind to different ligands, resulting in structural diversity (The RMSDs range from 0.2 to 4.6 Å). A total of 15 loop structures have been solved experimentally and these loops show an even higher level of structural diversity (RMSDs up to 33 Å). Three of these loops bind to magnesium ions. (Current prediction methods cannot distinguish calcium binding from magnesium binding. Some experimental evidence showed that calcium ions displace magnesium at the binding sites of calmodulin [25].) We modeled and scanned the other 15 loops and identified three calcium-binding sites (1GIH, 1GIJ and 1G5S). The RMSDs of the rebuilt loop of 1GIH to the 15 experimentally solved loop structures of others CDK2 range from 1 to 6 Å, suggesting that we have modeled reasonable loop conformations. A close-up view of Figure 4 shows that the rebuilt loop of 1GIH forms microenvironments suitable for calcium binding.

A structural homolog of 1GIH is the structure of rat TAO2 (a mitogen-activated protein kinase kinase kinase) in complex with MgATP (PDB ID: 1U5R). The PRA between 1GIH and 1U5R is 81%. 1GIH and 1U5R both possess a typical protein kinase two-domain architecture. The counterparts of unstructured loop 149-164 (1GIH) in 1U5R are residues 174-183, which constitute the activation loop of 1U5R. One calcium ion is observed near 181pSer. In the original report on 1U5R one calcium ion is observed to bind the activation loop, but the role of the calcium is not addressed[26].

We consider the binding of homologous proteins to calcium to be strong evidence of correct prediction. Table 5 and Figure 4 show calcium-binding sites in homologous holo structures demonstrate our method's robustness. Meanwhile such predictions provide novel functional insights into these proteins.

The remaining 78 predictions are novel sites in that their corresponding holo forms have not been experimentally tested (Additional file 1: Table S1). These sites are not recognized using the motif scan programs PROSITE and Pfam[27,28]. Some of these sites are found in the protein-membrane interface or near the binding area of ligands other than calcium. The candidate proteins that we scanned share SCOP folds families with known calcium-binding proteins. It is common that one protein may bind to multiple calcium ions. In the experimentally solved structures of 11 proteins for which we made predictions, calcium binding was experimentally observed at sites other than our predicted new sites. In these cases, our "hits" may represent additional new calcium sites or false positives. For the other 67 proteins, calcium binding has not been observed experimentally, and so our hits would be functionally new predictions of calcium binding. A total of 72 of these 78 loops contain at leaset one ASP or GLU residue (E or D), which are generally important residues for binding calcium, as they can provide amino acid side chains with negatively charged carboxyl groups. We discuss two examples for which independent evidence suggests that our predictions are correct.

**Figure 5:**
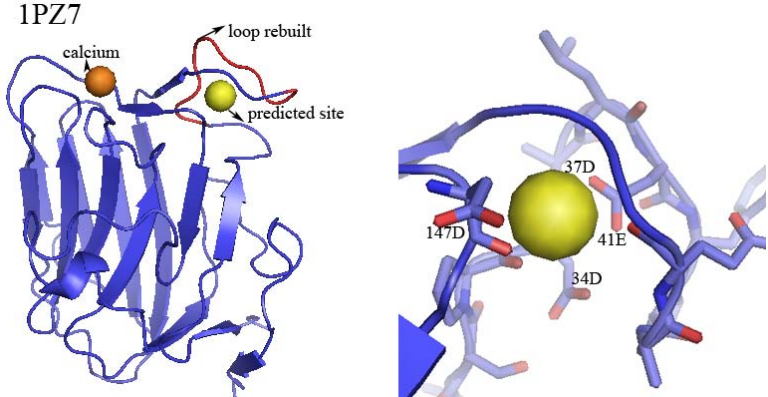
**Novel FEATURE predictions**. The structure of chicken argin G3 (PDB ID: 1PZ7) and the predicted site. The core structure of 1PZ7 is a beta-sandwich. The top edge of the beta-sandwich consists of four loops, which correspond to a versatile molecular recognition surface in argin. Calcium binding is observed near residues 137-144. Loop 32-40 (residues 32-40, sequence: SPDALDYPA) is unstructured in the original PDB structure. FEATURE predicts a binding site near loop 32-40 when the structure of this region was built using modeling methods. The predicted site is 13.52 Å away from the experimentally observed site; therefore it is a novel site. The close-up view (red for oxygen atoms) shows the close associations between the predicted site and two residues 34D, 37D, 41E and 147D. The overall fold of the two binding sites shares high homology with the C2 calcium-binding domain. The loops in the C2 domain often coordinate 2-3 calcium ions. This suggests our prediction of the second calcium-binding site is reasonable.

Figure 5 shows the structure of chicken argin G3 (PDB ID: 1PZ7). The argin induces the aggregation of acetylcholine receptors on the postsynaptic membranes of muscle cells. The core structure of argin is a beta-sandwich. The top edge of the beta-sandwich consists of four loops, forming a versatile molecular recognition surface in argin. Loop 32-40 (residues 32-40, sequence: SPDALDYPA) has been shown high mobility in NMR experiment. Disorder in loop 32-40 is observed in crystallography and is amplified in the absence of calcium. Using modeling methods, loop 32-40 is rebuilt. FEATURE identifies a calcium-binding site in 1PZ7 in the presence of the modeled structure of loop 32-40. Calcium is observed to rigidify this interface and thereby regulates the interaction between argin and acetylcholine receptors. Interestingly, calcium binding near residues 137-144 has been observed experimentally. The experimentally identified site is 13.52 Å away from our predicted site; therefore our prediction probably represents an additional new calcium site. The overall fold of the two binding sites shares high sequence similarity with the C2 calcium-binding domain. It is known that the loops in C2 domain often coordinate two or three calcium ions. We propose that argin G3 coordinates two calcium ions: one binds to loop 137-144 with high affinity, as observed experimentally; the other binds to loop 32-40 with lower affinity, as predicted by FEATURE.

**Figure 6:**
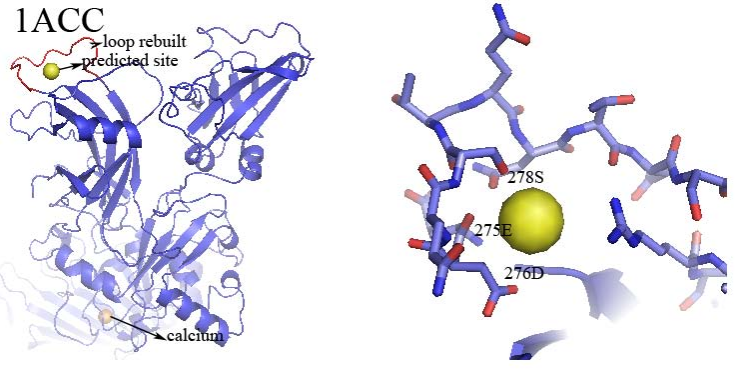
**Novel FEATURE predictions**. Structure of *bacillus anthraz *toxin protective antigen (PDB ID: 1ACC) and the predicted site. Loop 275-288 (residues 275-288, sequence: EDQSTQNTDSETRT) in 1ACC is unstructured. FEATURE predicts a calcium-binding site in 1ACC in the presence of the rebuilt structure for loop 275-288. The close-up view shows the close association between the predicted site and three residues 275E, 276D and 278S. The predicted site is part of domain 2 of 1ACC, which forms a beta-barrel with modified Greek-key topology, including a large flexible loop between strands. Calcium is predicted to bind in a cup-shaped depression formed by the loops of the beta-barrel structure. This beta-structure shares high structural homologies with the C2 calcium-binding domain, which often coordinates 2-3 calcium ions through its loops. Experimental evidence in the original work suggests that loop 275-288 is involved in membrane insertion. This corresponds to the fact that the C2 domain of many proteins plays important roles in calcium-dependent membrane binding. In summary, both structural and functional evidences show that calcium binding to the loops of the beta-structure is very likely. In addition, a calcium-binding site is observed in 1ACC, but it is 61.38 Å apart from our predicted novel site. The close up view (red for oxygen atoms) shows the close associations between the predicted sites and loop 275-288.

Figure 6 shows the structure of *bacillus anthraz *toxin protective antigen (PDB ID: 1ACC)[29]. FEATURE identifies a calcium-binding site in 1ACC with a modeled structure for a gap at residues 275-288 (sequence: EDQSTQNTDSETRT) was built using modeling methods. The predicted site is part of domain 2, which forms a beta-barrel with modified Greek-key topology, including a large flexible loop between strands. We predict that calcium binds in a cup-shaped depression formed by the loops of the beta-barrel structure. This beta-structure share high structural homologies with the C2 calcium-binding domain, which often coordinates 2-3 calcium ions through its loops. Experimental evidence in the original work also shows that the loop (residues 275-288) is involved in membrane insertion[29]. The C2 domain of many proteins plays important roles in calcium-dependent membrane binding. In summary, both structural and functional evidences show that calcium binding to the loops of the beta-structure is very likely. In addition, a calcium-binding site is observed in 1ACC, but it is 61.38 Å apart from our predicted novel site. Another structure of the anthrax toxin protective antigen (PDB ID: 1T6B) was found and the structure of region 275-288 is also missing. When we tested this site, we also identified it using our method of rebuilding and evaluating.

Coordinates of the predicted calcium-binding sites and the structures of the calcium-binding loops generated using modeling methods for all 256 predictions are available at: http://helix-web.stanford.edu/pubs/bmc-sb-liu/.

## Discussion

Our work uses the FEATURE program for recognizing calcium sites, but can be used with any similar structure-based method. FEATURE outperforms published methods in detecting divalent cation binding sites [8]. Glazer et al. compared FEATURE with another method for predicting ligand-binding sites (VALENCE). At a very low level of false positives (16 out of 22*10e3) FEATURE identified 21 out of 24 sites in holo structures and 16 out of 23 sites in apo structures; VALENCE identified 15 out of the 24 sites in the holo structures and zero out of 23 the sites in apo structures. The key contribution of this work is the combination of modeling and machine learning to recognize probable functions in disordered protein regions. Our method also produces a low-resolution structural model of the missing loops. Our goal is not to predict the structure of a binding site or loop with crystallographic accuracy, but to demonstrate that the modeled loop can adopt a conformation consistent with calcium binding. We first showed that FEATURE can recognize known ligand-binding loops that are rebuilt using modeling methods. We generated *de novo *loop structures using two separate strategies. The RMSDs between the 100 rebuilt loops and the corresponding natural apo loop structure range from 0.7 to 6.5 Å; and those to the corresponding natural holo structure range from 0.8 to 5.6 Å. The rebuilt loops show good structural diversity. We demonstrated that a cutoff of 50 has very low false-positive rate, a critical feature for prediction. Since biologists may pursue a prediction with experiments, it is generally more important to have a low false positive rate than a low false negative rate. On the other hand, false positives can lead to wasted experimental resources. False negatives are not typically tested and so although regrettable, do not generally consume resources. The trade off depends on the particular goals of the investigator, and so the cutoffs can be changed to accommodate different priorities for sensitivity and specificity.

At the cutoff of 50, the RMSDs between the FEATURE-recognized loops and the corresponding natural apo loop structure range from 1.3 to 4.8 Å; and those to the corresponding natural holo structure range from 1.3 to 4.7 Å. Thus, the modeling protocol produces diverse loops, and then FEATURE identifies the subset whose configuration is compatible with calcium binding. Table 2 shows two proteins (1DVIB277 and 1K94_998) where the experimental apo structure shows strong calcium binding signal with FEATURE, but none of the rebuilt loops show strong signal. Thus, even though the model building generally works, it does not always generate variations that are compatible with calcium binding.

We went on to show that we can combine FEATURE with modeling methods to find binding sites that are not completely resolved experimentally (they are unstructured or disordered in the crystal structure). We applied this procedure to the candidate calcium-binding proteins for which experimentally solved structures are incomplete, resulting in 256 novel predictions of calcium-binding sites. As discussed above, we selected a cutoff score that increases the likelihood that these calcium-binding predictions are correct. Some of our predictions may nonetheless be false positives, but our analysis suggests that they are very likely to be functionally interesting regions of these proteins. For example, they may bind other cations or positively charged ligands. Magnesium binding is very difficult to distinguish from calcium binding using "low resolution" methods such as FEATURE, where features are averaged in shells of thickness 1.25 Angstroms. These sites display a variety of structural characters and have a broad range of functions, providing novel insights into the studies of calcium-binding proteins.

To further test the usefulness of our protocol, we have applied it to CASP8 targets http://predictioncenter.org/casp8/. There is only one calcium-binding protein in the 128 CASP8 targets, C-terminal domain of probable hemolysin from *chromobacterium violaceum *(PDBID: 3DED). We scanned 24 loops in 3DED, including six calcium-binding loops and 18 loops as negative controls. FEATURE successfully identifies the six calcium-binding loops. For each positive prediction, we found multiple high FEATURE scores in both crystallographic loops as well as our modeled loops. These high scores are distributed over a relatively large volume, most likely indicating more than one calcium ion in these loops. In fact, each pair of loops binds 2 calcium ions. None of the 18 loops (negative control) are predicted as false positive. These results demonstrate that our procedure can identify calcium-binding sites successfully even when the structures of binding-loops are rebuilt using modeling methods.

Our prediction strategy focused on proteins with electron density gaps that are in SCOP families that bind calcium. We decided to focus on these to limit the number of gaps that we modeled and evaluated for this study, and because it increased the chance that our predictions would be biologically plausible and interpretable. It would certainly be possible to predict calcium-binding loops for all unstructured loops. Interestingly, only 11 of the 78 highest hits were in proteins that already had resolved binding sites. These 11 predictions are thus for an additional calcium site. This is not at all unusual, as many proteins bind multiple calcium ions. The remaining top 67 predictions are all novel and would represent the first calcium-binding site in these structures.

The most well known calcium-binding site is the EF-hand sequence motif [30]. We scanned 500 different SCOP fold families known to bind calcium. About 2% of structures (about 29000 structures) in these 500 families contain EF-hand motif. Our study focused on 2495 structures with regions of poor (or missing) electron density. Only 0.08% (total 19 structures) of the 2485 structures contain EF-hand motifs. Furthermore, only three loops that we modeled and scanned are within the EF-hand motifs. We successfully recognize the calcium-binding activity of these three loops: they are 1SL9, 1U5R and 1DF0 (homolog of 1U5R). The paucity of EF-hand missing protein loops suggests that they tend to be stable, even in the absence of calcium. Indeed, we find that both the apo and holo states of EF-hand form ordered structures; and so experimental 3D structures of both forms are usually available. The remaining (non-EF-hand) sites generally are not associated with good sequence motifs, and seem to show higher structural flexibility

We have observed that a considerable number of predictions adopt anti-parallel beta structures, but in different topologies, including beta-barrel, beta-sandwich and Greek key. These proteins display a wide range of function roles, including photosynthesis (1DBZ), aggregation of acetylcholine receptors (1PZ7), membrane insertion as a responsible for anthrax (1ACC). For these proteins, calcium ions are often predicted to bind the loops between beta-strands. These structures show structural homologies with the C2 domains, which form an eight-stranded anti-parallel beta-sandwich consisting of a pair of four-stranded beta-sheets. The C2 domain is often involved in calcium-dependent membrane targeting. Calcium ions often bind in an indentation formed by the first and final loops of the membrane binding face of the C2 domain. Newton et al proposed a model for calcium-dependent membrane binding by the C2 domain: calcium binding induces a conformational change in the C2 domain in order to expose functional groups responsible for membrane binding [31]. In these predicted sites found in the anti-parallel beta structures, loops are not observed in crystal structures due to their high flexibility. We proposed that calcium binding to these loops induces conformational changes, which contribute to the functional roles of the loops.

We note that 61 proteins of our 78 novel predictions bind to other ligands in addition to metal ions. In those cases, it is possible that calcium plays a role in regulating the binding of other ligands, as has been observed in some proteins[32,33]. For example, calcium induces a conformational change in the ligand-binding site of the LDL receptor and maintains the cysteine-rich regions in a more folded, native state. In the absence of calcium, the LDL receptor does not have the proper conformation for ligand binding. Consequently, only when the LDL receptor is exposed to calcium, it is competent to bind the ligand [33]. In our predictions, we have predicted that calcium binding regulates the binding of 1PU or other consequent activity changes to the cell division protein kinase 2 (1GIH) (see the Results). Our prediction has been confirmed by the observation of its homologous structure 1U5R. A similar role of calcium binding is observed in one of the 78 novel predictions, mycobacterium tuberculosis RecA (PDB ID: 1G18). We therefore propose that these 61 proteins' activity may be regulated by calcium binding via allosteric effects.

## Conclusion

We modeled and identified calcium-binding sites for which experimentally solved  structures are not available because they have high flexibility and lack ordered  structures. Combining a machine learning site-recognition algorithm (FEATURE) with a *de novo *loop modeling technique enabled us to capture binding sites not apparent in a validation set of 20 apo structures. We not only predict the calcium binding function, but produce at least one structural conformation to support this prediction. The idea of improving models using functional information is attractive, and may be applicable to other structure modeling efforts, when functional information is available. From the 2745 structural gaps in experimental structures, we made 102 non-redundant predictions of calcium-binding sites that achieve high FEATURE scores. In these 102 predictions, ten predicted sites are confirmed by experimental evidence; 14 predicted are supported by indirect experimental evidence; 78 sites are novel predictions. In the 78 novel predictions, a large number of predicted sites adopt anti-parallel beta structures, which share structural similarity with the calcium-binding C2 domain. A total of 61 of our 78 predictions are in proteins that bind to other ligands, suggesting a role of calcium in regulating ligand binding in these proteins.

## Methods

### Selection of validation dataset

Babor M et al created a non-redundant dataset of apo-holo pairs of metal-binding proteins[9]. Using this dataset, we selected apo-holo pairs of calcium-binding proteins in which the primary atomic contacts are between the calcium the protein structure (and not via water bridges). Thus, we filtered out sites where binding is mediated by water molecules. The filter counts atoms within 2.5 Å of a calcium-binding site (close contacts) and retains sites for which the number of close contact atoms from protein structures is three or more and from water molecules is less than two. The dataset of positive sites consists of the calcium-binding loops in apo and holo structures. The dataset of nonsites consists of a randomly selected loop (excluding the calcium-binding loop) in each of the holo structure and the counterpart in the apo structure.

The RMSDs were calculated between the apo and the holo structures by using the program TM-score which compares two structures based on their given residue equivalency[34]. The RMSDs between loops in the apo and the holo forms are provided in Table 1.

### Construction of loop structures

Structures of calcium-binding loops were generated using programs in the RAMP software suite [12-14]. These programs are *mcgen_exhaustive_loop *and *mcgen_semfold_loop*, which use *de novo *modeling method to build loop conformations for a given region in protein structures. The former generates structures by exhaustively enumerating all possible main chain structures using a 14-state *phi/psi *model and selecting the best ones using a residue-specific all-atom conditional probability discriminatory function. The latter one is based on inserting small (usually three-residue) fragments randomly and uses a Monte Carlo with simulated annealing procedure to find the best combinations of these fragments.

### FEATURE scan

Calcium-binding sites were predicted using FEATURE. A complete description of FEATURE can be found in the original paper and a web server of FEATURE is also provided in http://feature.stanford.edu/webfeature[5]. In summary, we make observations of 66 physical-chemical properties on a dataset of experimentally determined structures containing calcium-binding sites. We then compile a conditional probability model of the distribution of these properties in calcium-binding sites. This model divides the space around a site into six concentric shells of 1.25 Å thickness. Given a site in a particular structure, the values of 66 properties within a certain distance cutoff are summed to yield the total FEATURE score to evaluate the probability of its likelihood of being a calcium-binging site. A FEATURE score of 50 has a high sensitivity and specificity for calcium binding [15].

In this work, we defined a cubic grid of 0.5 Å superimposed upon the existing or rebuilt calcium-binding loop. The grid extends 5 Å beyond the extreme Cartesian coordinates of the atoms in each loop structure. Grid points with few or no atoms are eliminated. For each grid point, we calculated its likelihood of being a calcium-binding site. We chose the grid point with highest FEATURE score as the final predicted binding site.

### Comparison of FEATURE performance on the apo structure and the apo structure with loop reconstructed

For each calcium-binding site in the validation dataset, we built two structures (in addition to the crystallographic holo and apo structures): the apo structure with its calcium-binding loop removed, named "apo-gap" (this simulates a crystal structure with missing density); and the apo structure with its loop built using modeling, named "apo-loop" (this simulates a loop that is rebuilt because the density is missing). We applied a FEATURE grid scan to four structures: the holo structure, the apo structure, the apo-gap and the apo-loop. We then compared the number of true positive sites recognized by FEATURE at different conditions. We also applied the procedure to nonsites as a negative control.

### Prepare candidate dataset for prediction

We created a list of SCOP families for which at least one member binds calcium. All solved 3D structures containing one or more calcium ions were downloaded from the Protein Data Bank (PDB). 3352 of these structures were determined by X-ray diffraction. The 3352 structures were matched against the SCOP database, resulting in a total of 500 unique SCOP fold families [35]. We then downloaded all 15404 structures from PDB which were categorized into these 500 SCOP fold families according to ASTRAL SCOP 1.73 database[35]. We further filtered PDB structure chains using the following criteria: (1) The structure chain was determined by X-ray diffraction; (2) Full sequence information is available in that PDB structure file; (3) The structure chain is not completely determined and the structural gap is more than 4 residues and less than 17 residues. This procedure resulted in a candidate dataset consisting of 2030 PDB chains containing 2745 structural gaps.

### Prediction of novel calcium-binding sites

For each structural gap in the candidate dataset, 100 all-atom loop structures were generated using programs from the RAMP suite as described above. For each of the 100 loop structures, we built a scan grid surrounding all the loops. For each loop, we applied FEATURE to each point in the grid to evaluate potential calcium binding positions around the loop. When the FEATURE score of the gap (no electron density) is lower than 50 and that of the corresponding built loop is higher than 50, we consider the point to be a predicted calcium-binding site and record the associated loop conformation coordinates. For each loop, if more than one point around a particular loop scores more than 50, we select the point with the highest FEATURE score. If more than one loop in a set of rebuilt loop conformational candidates scores above 50, we select the one with the highest FEATURE score. Thus for each structural gap, we collect a maximum of one grid point (corresponding to the predicted calcium-binding site) and one corresponding loop conformation. To avoid redundant predictions across closely related protein homologs, we filter out PDB chains with the same sequences.

### Validation of predictions

For each prediction, we first attempted to validate it by examining its homologous structures, reasoning that if a homologous structure shows calcium binding in a similar location, it would be strong, though indirect, evidence that our prediction is correct. For this comparison, we used structures in the same SCOP fold family. We generated structural alignments between FEATURE predictions and their corresponding homologous candidates using the software program MAMMOTH[19]. We calculated the percentage of residues aligned (PRA) and the percentage of identical residues (PID) based on the MAMMOTH alignment, and recorded the distances between the calcium-binding loop in the homologous counterpart and calcium ions (CaDis). By using different cutoffs of PRA, PID and CaDis, the predicted binding sites were divided into groups. The cutoff for CaDis was set to 7.5 Å (the radius of the FEATURE model for a calcium site). We further analyzed predictions in each group by visual inspection.

All figures were prepared with MacPYMOL[36].

## Authors' contributions

TL carried out the computational analysis. RBA participated in designing the study and preparing the manuscript.

## Supplementary Material

Additional file 1 **Table S1**. Table displaying 78 novel FEATURE predictions Click here for file
