# Supplementary material for: Prediction of calcium-binding sites by combining loop-modeling with machine learning
Source: BMC Struct Biol. 2009 Dec 11;9:72. doi: 10.1186/1472-6807-9-72 (PMC2808310; doi:10.1186/1472-6807-9-72)
Supplement: Additional file 1 — Table S1. Table displaying 78 novel FEATURE predictions [file 1472-6807-9-72-S1.doc]

Table S1. 78 novel FEATURE predictions.

| Protein | Loop | | | FEATURE Score | | | Ligands observed |
| --- | --- | --- | --- | --- | --- | --- | --- |
|  | Start | End | Sequence | Gap | | Loop | experimentally |
| 1TFZ_A | 230 | 242 | A**E**FTA**DD**VGTA**E**S | 43.48 | | 89.37 | FE,869 |
| 1F5N_A | 157 | 165 | SP**DE**N**E**N**E**V | 13.29 | | 78.18 | MG,GNP |
| 1B7T_A* | 199 | 212 | AVKKK**DEE**AS**D**KK**E** | -15.76 | | 77.63 | CA,MG,ADP |
| 1G5C_B* | 12 | 24 | **D**FRFR**D**LS**D**LKHS | 37.57 | | 77.09 | CA,ZN,EPE |
| 1JRG_B | 213 | 225 | GHN**D**TNSAQ**D**KGK | -31.35 | | 75.49 | SO4 |
| 1DBZ_D | 152 | 164 | ESLP**D**FG**DD**S**DD**N | -34.69 | | 73.70 | none |
| 1LL1_A | 22 | 33 | IG**D**G**D**KHKHS**D**R | -12.06 | | 73.48 | CL,CU |
| 1TKH_A* | 198 | 202 | **E**G**D**GR | 47.21 | | 71.42 | CA,ZN,DPN |
| 1PVO_A | 147 | 152 | M**E**RGNG | 2.312 | | 70.72 | ANP |
| 2BDY_A | 31 | 39 | SYI**D**GRIV**E** | 16.21 | | 70.32 | NA,TYS,UNB |
| 1G18_A | 196 | 211 | LR**D**KIGVMFGSP**E**TTT | 25.23 | | 69.02 | ADP,ALF |
| 1Q1Q_A | 95 | 103 | AFSLP**D**QYS | 47.68 | | 68.52 | NA,A3P,NHE |
| 3MAN_A | 228 | 234 | H**D**HS**D**GN | -3.20 | | 67.30 | BMA,MAN |
| 1PZ7_B* | 32 | 40 | SP**D**AL**D**YPA | 7.98 | | 66.65 | CA |
| 1ACC_A* | 275 | 288 | **ED**QSTQNT**D**S**E**TRT | 3.74 | | 66.59 | CA |
| 2G28_A | 400 | 414 | A**E**GKNIAHQVKKMNM | 33.57 | | 65.50 | MG,TDK |
| 1M80_A | 308 | 321 | VRGTRG**E**HT**E**S**E**GG | -23.85 | | 64.28 | none |
| 2EFX_D | 207 | 220 | AA**DDE**NPQW**D**VSGA | -45.84 | | 64.28 | BA,NFA |
| 1UKH_A | 336 | 349 | KIP**D**KQL**DE**R**E**HTI | 10.08 | | 63.15 | none |
| 1P1Z_A | 34 | 45 | VRF**D**S**D**A**E**NPRY | -13.31 | | 62.81 | none |
| 2SHP_B | 235 | 246 | KLA**E**TT**D**KVKQG | -16.31 | | 62.05 | CAT |
| 1QQE_A | 227 | 237 | S**ED**PNFA**D**SR**E** | -42.65 | | 61.78 | none |
| 1GXB_C | 79 | 84 | GTGG**D**G | 10.15 | | 61.38 | MG,POP |
| 1SQI_A | 212 | 224 | V**DD**TQVHT**E**YSSL | 21.60 | | 61.14 | FE,869 |
| 1A3C_A | 73 | 82 | R**DD**LSKKTSN | -19.88 | | 60.96 | SM,SO4 |
| 2I3S_E | 224 | 233 | **DD**QG**DD**YNSS | 5.01 | | 60.63 | none |
| 1GY8_B | 235 | 249 | AP**D**QRLTIH**ED**AST**D** | 13.94 | | 60.46 | NAD,UDP |
| 1A7J_A | 99 | 106 | VH**DD**A**E**AA | 14.41 | | 59.93 | SO4 |
| 2P9I_A* | 39 | 53 | **E**SAKVG**D**QAQRRVMK | -21.53 | | 59.58 | CA,ADP |
| 1YG2_A | 58 | 68 | **E**PQ**E**GKP**D**RKV | -27.09 | | 58.91 | none |
| 1VFZ_A | 292 | 303 | PNKNKKKKKT**D**F | -20.03 | | 57.75 | MG,ADP,VO4 |
| 1BQG_A | 98 | 111 | A**D**R**D**VGGRGLQTF**D** | -57.42 | | 57.67 | None |
| 1S7H_B | 95 | 101 | YLSKG**D**K | 25.04 | | 57.56 | None |
| 1V04_A* | 71 | 79 | YPGIMSF**D**P**D** | -15.06 | | 57.40 | CA, PO4 |
| 1TFZ_A | 172 | 180 | KA**ED**T**E**KS**E** | 10.32 | | 57.37 | FE,869 |
| 1SUL_A | 50 | 60 | ARTSSKPGKTQ | 20.12 | | 57.17 | none |
| 1G20_G* | 186 | 191 | SRNT**D**R | -3.82 | | 56.79 | CA,CFM,CLF,HCA,SF4 |
| 1FIE_A | 29 | 44 | V**E**LQGVVPRGVNLQ**E**F | 28.83 | | 56.70 | none |
| 1FG9_D | 141 | 148 | **E**V**D**Y**D**P**E**T | -14.04 | | 56.60 | none |
| 2FBK_A | 120 | 128 | **EDE**R**D**RRSA | -13.55 | | 56.45 | CL |
| 2E1R_A | 50 | 65 | KAG**E**ARFT**D**TRK**DE**Q**E** | -47.09 | | 56.40 | GDP,SOD |
| 1SP3_A | 146 | 153 | GGGG**D**AVK | -27.49 | | 56.27 | HEM,SCN |
| 1OZT_L | 66 | 79 | PLSRKHGGPK**DEE**R | -12.95 | | 55.71 | none |
| 1AVC_A* | 323 | 330 | G**DDD**AAGQ | 5.57 | | 55.51 | CA |
| 1SR5_A | 354 | 360 | IVA**E**GR**D** | 11.91 | | 55.21 | NAG,NT1,NT2 |
| 1JAL_B | 108 | 122 | **E**N**DD**IVHVAGKI**D**PL | 7.21 | | 54.86 | none |
| 1SMS_A | 305 | 315 | **ED**VATAGKTTF | 2.65 | | 54.61 | HG |
| 1LS1_A | 271 | 278 | GVS**E**KP**E**G | -4.59 | | 54.33 | MG,OXY |
| 1FMU_A | 161 | 166 | **D**T**E**NGG | 44.77 | | 54.22 | MAN,NAG,NDG |
| 1FCM_A | 280 | 287 | IINGS**D**NK | 6.67 | | 54.11 | CXN |
| 1RFQ_A | 40 | 51 | HQGVMVGMGQK**D** | 12.08 | | 53.94 | MG,ATP,LAR |
| 1RQX_D | 129 | 139 | VP**D**GF**D**IGFRR | -36.71 | | 53.82 | MLP,PLP |
| 1VFW_A | 254 | 268 | RA**D**STGAKGTRLK**E**G | -38.071 | | 53.80 | MG,ANP |
| 2GUF_A | 573 | 582 | Y**E**TVYGYQTA | 36.79 | | 53.77 | FMT,MPD,MPG |
| 1P1Z_A | 103 | 111 | VGS**D**GRLLR | -6.40 | | 53.71 | none |
| 2BOD_X | 80 | 87 | CGNHSSGG | 20.12 | | 53.66 | BGC,MGL,SGC |
| 2REN_A | 165 | 171 | **D**S**E**NSQS | 24.45 | | 53.59 | NAG |
| 1P2M_C | 10 | 16 | LSGLSRI | -7.69 | | 53.45 | SO4 |
| 1PBG_A | 314 | 325 | IHNGKG**E**KGSSK | -7.38 | | 53.25 | SO4 |
| 1JK0_B | 90 | 100 | ISS**D**NLVNKYL | 4.09 | | 53.02 | ZN |
| 1B4N_B* | 453 | 462 | **E**G**E**KA**E**KV**E**Y | -3.89 | | 52.83 | CA,GUA,PTE,SF4 |
| 1NQ9_I | 133 | 137 | KANKS | 13.45 | | 52.38 | NAG,NTP |
| 1AKM_A | 234 | 246 | VSMG**E**AK**E**KWA**E**R | 18.27 | | 52.18 | none |
| 1PR3_A | 40 | 55 | AGQKAPVFGGK**D**AG**D**L | -53.76 | | 52.10 | PO4 |
| 2D1Y_A | 192 | 201 | ALSP**D**P**E**RTR | -16.72 | | 51.94 | NAD |
| 1TQD_A | 189 | 202 | ITN**E**A**D**GKPQTF**D**K | -55.99 | | 51.93 | MES,SO4 |
| 1MDZ_A | 220 | 232 | **D**AW**D**RQSGGRAPQ | -11.61 | | 51.90 | DCS,PLP |
| 2GYS_A | 342 | 351 | TMKMRY**E**HI**D** | -7.26 | | 51.41 | BMA,FUC,NAG,NDG |
| 1P4A_B | 162 | 167 | **D**NKVT**E** | -4.86 | | 51.40 | PCP |
| 1J8Y_F | 108 | 113 | GTGKAT | 17.47 | | 51.35 | none |
| 1T67_A | 84 | 92 | Q**E**G**DDD**HP**D** | -19.75 | | 51.22 | NA,ZN,B3N |
| 2ACE_A | 484 | 490 | **E**PHSQ**E**S | 13.23 | | 50.85 | ACH |
| 1DCU_D | 66 | 76 | GTQGAVNIQG**E** | | 35.00 | 50.82 | none |
| 1BM3_H | 99 | 111 | HPFYRY**D**GGNYYA | | 10.50 | 50.75 | none |
| 1EF8_C | 67 | 79 | **D**IH**E**LPSGGR**D**PL | | 5.75 | 50.43 | NI |
| 1G20_G* | 49 | 55 | LHSKAQN | | 42.63 | 50.15 | CA,CFM,CLF,HCA,SF4 |
| 1SR6_A* | 730 | 734 | QGFV**D** | | 13.42 | 50.12 | CA,MG,SO4 |
| 1PXO_A | 36 | 41 | RL**D**T**E**T | | -0.91 | 50.02 | CK7 |

Protein name is PDB code followed by the chain identifier. We build structures for gaps of which 3D structures are not present in the original PDB files. Information of these structures, referred as “loop“, is given in column 2-4. Column 5 and 6 list FEATURE scores at the predicted sites in the structures where loop structures are missing (Gap) and in the rebuilt loop (Loop). The ligands observed experimentally are listed in column 7 and the ligand IDs are from PDB. 11 of these 78 proteins bind to calcium ions at sites other than the predicted sites (indicated with a ‘*’).
